# Cognitive Models for Machine Theory of Mind

**DOI:** 10.1111/tops.12773

**Published:** 2024-12-01

**Authors:** Christian Lebiere, Peter Pirolli, Matthew Johnson, Michael Martin, Donald Morrison

**Affiliations:** ^1^ Department of Psychology Carnegie Mellon University; ^2^ Institute for Human & Machine Cognition Pensacola

**Keywords:** Human–machine teaming, Theory of mind, Cognitive models, Intelligent agents, Personalization, Model tracing, Instance‐based learning, ACT‐R

## Abstract

Some of the required characteristics for a true machine theory of mind (MToM) include the ability to (1) reproduce the full diversity of human thought and behavior, (2) develop a personalized model of an individual with very limited data, and (3) provide an explanation for behavioral predictions grounded in the cognitive processes of the individual. We propose that a certain class of cognitive models provide an approach that is well suited to meeting those requirements. Being grounded in a mechanistic framework like a cognitive architecture such as ACT‐R naturally fulfills the third requirement by mapping behavior to cognitive mechanisms. Exploiting a modeling paradigm such as instance‐based learning accounts for the first requirement by reflecting variations in individual experience into a diversity of behavior. Mechanisms such as knowledge tracing and model tracing allow a specific run of the cognitive model to be aligned with a given individual behavior trace, fulfilling the second requirement. We illustrate these principles with a cognitive model of decision‐making in a search and rescue task in the Minecraft simulation environment. We demonstrate that cognitive models personalized to individual human players can provide the MToM capability to optimize artificial intelligence agents by diagnosing the underlying causes of observed human behavior, projecting the future effects of potential interventions, and managing the adaptive process of shaping human behavior. Examples of the inputs provided by such analytic cognitive agents include predictions of cognitive load, probability of error, estimates of player self‐efficacy, and trust calibration. Finally, we discuss implications for future research and applications to collective human–machine intelligence.

## Abstract 2

We propose three criteria for a machine theory of mind and argue that personalized instance‐based cognitive models can satisfy those requirements. We demonstrate those capabilities in a simulated search and rescue task by showing that computational cognitive models can diagnose the cause of observed behaviors and adaptively optimize human–machine interaction.

## 1 Introduction

Investment in artificial intelligence (AI) is at an all‐time high. New technologies such as deep learning seem to be breaking new barriers on a regular basis, learning to master video games, beating the best human players at games of strategy, and creating seemingly original graphical and textual works. However, a recent report by the National Academies (Endsley et al., 2022) points out some limitations of AI such as brittleness and biases and emphasizes the need for human–AI teaming for the foreseeable future. Requirements include the need for human teammates to understand, predict, trust, and be able to control AI. That view is also shared by another report from a recent workshop on human–machine teaming (Laird, Ranganath, & Gershman, 2020). But it also emphasizes that while humans need to develop an accurate model of the machines with which they interact, the converse is true as well. Machines also need to include a model of their human teammates to adapt to their preferences, capabilities, and limitations.

To enable true human–machine teaming, we argue that some of the required characteristics for a machine theory of mind (MToM) include the ability to (1) reproduce the full diversity of human thought and behavior, (2) develop a personalized model of an individual with very limited data, and (3) provide an explanation for behavioral predictions grounded in the cognitive processes of the individual. We propose that a certain class of cognitive models provide an approach that is well suited to meeting those requirements. Being grounded in a first‐principles framework like a cognitive architecture, informed and constrained by a unified theory of cognition, naturally fulfills the third requirement by mapping behavior to cognitive mechanisms. Exploiting a modeling paradigm such as instance‐based learning (IBL) accounts for the first requirement by reflecting variations in individual experience into a diversity of behavior. Mechanisms such as knowledge tracing and model tracing allow a specific run of the cognitive model to be aligned with a given individual behavior trace, fulfilling the second requirement.

While data‐driven machine learning techniques currently dominate AI, we argue that personalized, first‐principles cognitive models based on theoretical frameworks provide unique advantages for human–machine teaming. They provide an account of the learning processes of human teammates, not just their average performance over time. They can be quickly personalized to individual human teammates with a small fraction of the data needed by machine learning techniques (Lebiere, Gray, Salvucci, & West, 2003). They provide a predictive, generative model of the human response to novel interventions for which no data are available (Cranford, Aggarwal, et al., 2020). They can be used in several ways, including run in Monte Carlo mode as computational oracles, abstracted into analytically tractable closed‐form solutions, or used as generators of training data for machine learning models (Sycara et al., 2015). Finally, they can provide the means of introspecting in the workings of the model in a way that black box models do not allow, leading to improved predictability and ultimately trust (Cranford, Somers, Mitsopoulos, & Lebiere, 2020; Mitsopoulos et al., 2021).

The outline of this paper is as follows. The next section provides a general introduction to the ACT‐R cognitive architecture, the IBL methodology for developing cognitive models, and some of the advantages of that approach. The following section discusses related work on other approaches to modeling the theory of mind. The setting for this work, a synthetic search and rescue task in the Minecraft environment, is briefly introduced. A cognitive model of the task is then described in detail and results of its personalized fit to individuals are presented. The following section introduces a measure of cognitive load used to inform intelligent agents and presents results validating that measure and the resulting interventions designed to improve teamwork. A final discussion section points to future research directions, including how to combine individual cognitive models with a framework to capture interdependencies between tasks and teammates to provide a constrained representation of cognitive teamwork.

## 2 Personalized models using cognitive architectures

Cognitive architectures are computational implementations of unified theories of cognition (Newell, 1990). Cognitive architectures include mechanisms and representations abstracted from human behavior, arranged as fine‐grained interactions between functional modules that reflect the structure and operation of the human brain. A wide variety of cognitive architectures have been proposed over the last five decades since the concept was introduced as a unification of functionality‐specific models to provide an integrated account of human cognition (Newell, 1973). Recently, an attempt has been made to extract an emerging consensus regarding the central structures and processes of cognitive architectures in the form of a common model of cognition (CMC), initially called the standard model of the mind (Laird, Lebiere, & Rosenbloom, 2017).

ACT‐R (Anderson & Lebiere, 1998; Anderson et al., 2004) provides a computational implementation of the CMC informed by the rational analysis of cognition (Anderson, 1990) that assumes that our cognitive mechanisms and representations have adapted to the statistical structure of our environment. This assumption enables the development of models based on the cognitive architecture that abstracts over details of our personal environment to generate behaviors that respond to the overall regularities of our information landscape.

The ACT‐R theory has evolved to address a wide variety of experimental results on problem‐solving, decision‐making, memory, learning, cognitive skill acquisition, perception, and attention, as well as the fine‐grained time course of neural processes. The theory has been applied to a variety of domains including computer tutoring systems (Anderson, Corbett, Koedinger, & Pelletier, 1995), human–computer interaction (John, Prevas, Salvucci, & Koedinger, 2004), and language learning (Pavlik & Anderson, 2008). ACT‐R is implemented as a simulation environment that supports the development of cognitive models.^1^

**Fig. 1:**
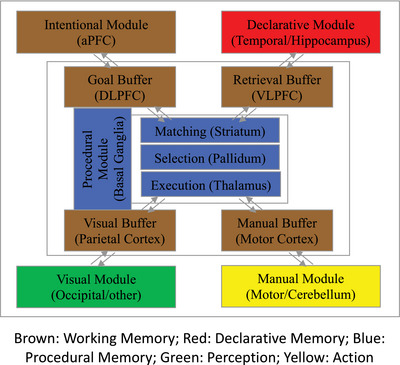
ACT‐R cognitive architecture.

ACT‐R (Fig. 1) is composed of modules, processing different kinds of content, which are coordinated through a centralized procedural module. Each module corresponds to a brain region. Each module is assumed to access and deposit information into one or more associated buffers, and the central procedural module can only respond to the contents of the buffers. The procedural module matches the contents of those buffers and coordinates the activity of the modules using production rules, which are pairs of conditions and associated actions. Neurally, a production rule is a formal specification of the flow of information from buffers in the cortex to the basal ganglia and back again. Productions have a utility value that is used to select the single rule that is executed at any point in time.

Knowledge and experience in the declarative module are represented as sets of attribute–value pairs called chunks. Chunks have activation levels that determine the probability and time course of chunk retrieval into a buffer. Chunk activations are real‐valued quantities produced by subsymbolic mechanisms in ACT‐R. These subsymbolic mechanisms reflect neural‐like processes that determine the time course and probability of cognitive activity and behavioral performance. The dynamics of declarative memory retrieval and production selection are determined by these subsymbolic mechanisms.

Developing cognitive models of a specific task, even when leveraging mechanisms from a cognitive architecture, still represents a significant endeavor. The IBL modeling approach addresses that issue by assuming that performance is primarily based on experiences in the task environment (Gonzalez et al., 2003). This relieves the modeler of the burden of designing, implementing, and validating specific task strategies. Rather, strategies emerge gradually from the accumulation of experience, constrained by the use of cognitive mechanisms, especially of long‐term memory, to store and access that knowledge and use it to generate expectations that are the main basis of decisions.

IBL has been used to model decision‐making processes across several domains (Hertwig, 2015). Some applications include repeated binary‐choice decisions (Gonzalez & Dutt, 2011; Lebiere, Gonzalez, & Martin, 2007), multi‐person/multi‐choice games such as backgammon (Sanner, Anderson, Lebiere, & Lovett, 2000), rock‐paper‐scissors (West & Lebiere, 2001), and Stackelberg security games (Abbasi et al., 2016), dynamic environments such as social dilemmas (Gonzalez, Ben‐Asher, Martin, & Dutt, 2015; Juvina, Saleem, Martin, Gonzalez, & Lebiere, 2013; Lebiere, Wallach, & West, 2000), supply chain management (Gonzalez & Lebiere, 2005), and resource allocation (Gonzalez et al., 2003) and automated detection systems (Thomson, Cranford, & Lebiere, 2021; Thomson, Lebiere, Bennati, Shakarian, & Nunes, 2015). These examples highlight the theory's generalizability in modeling decisions from experience.

Modeling a specific individual requires aligning the experiences of a model with that of an individual. For this reason, IBL models can combine naturally with model‐tracing techniques to tailor systems to an individual. By tracking an individual's experience, an IBL model can make accurate predictions of their performance and those predictions can then be used to adapt and personalize human–machine interactions. Model tracing is a technique commonly used to adjust feedback provided to the student in intelligent tutoring systems (Anderson et al., 1995). Model tracing can be used to synchronize an IBL model with the human's observed actions and experience. After each trial, the instance saved in memory that represents the model's decision and outcome is changed to reflect the human's action and outcome (i.e., the action and outcome slots of the current instance are changed to match the human's). Therefore, on the next trial, the model makes predictions based on the exact experience of the human and not on what it would have done based on its own past instances. As shown in Cranford, Gonzalez, et al. (2020), with more experiences to draw from, IBL models make more accurate predictions of a particular human's actions. Cognitive twins can be developed to reproduce the preferences of human users with only a small sample of their experiences (Somers, Oltramari, & Lebiere, 2020).

While tracing a user's decisions is an effective method for aligning a model against an individual, there are additional methods that can be used to further personalize the model. One method that has shown to be useful is by introspecting the cognitive model to learn what contextual features are most impactful on an individual's decisions. By examining the cognitive salience of features in an IBL model (Mitsopoulos et al., 2021; Somers, Mitsopoulos, Lebiere, & Thomson, 2019), we can learn what features are most important for an individual's decision‐making and use this information to further adapt a system or even the cognitive model itself.

## 3 Related work

Theory of mind (ToM) is a very general topic covering a broad range of scientific and technical issues. In this paper, we are primarily concerned with a general approach to representing the beliefs of agents and their impact on decision‐making. Specific aspects include an efficient learning process of an accurate model of individual differences, the development of a representation of knowledge that also captures implications for its processing such as workload, a model that enables cognitively plausible strategies for optimizing performance including minimizing workload, and the generation of measures that can be used by artificial agents to optimize human–machine interaction. A comprehensive survey would be beyond the scope of this paper, but we highlight here some of the most directly relevant work from a cognitive perspective.

One of the central issues in the field of theory of mind is modeling the development of the ability to reason about false beliefs in others. Triona, Masnick, and Morris (2019) presented an ACT‐R model that passes the false belief task. However, the focus of that model is on hand‐engineered productions for answering questions specific to the task and is not generally concerned with belief representation or the resulting constraints of cognitive mechanisms. Arslan, Taatgen, and Verbrugge (2013) also developed an ACT‐R model of the false belief task using a combination of rule‐based and simulation approaches. They modeled the gradual development of reasoning about false beliefs of others by using the activation of declarative knowledge instead of the utility learning of production rules. Hiatt and Trafton (2010) developed perhaps the most general cognitive model of the theory of mind in the context of reasoning about beliefs, desires, and intentions. That model instantiates in the ACT‐R cognitive architecture and integrates three theories of ToM: theory–theory, simulation theory, and mechanism theory. The focus of these models is distinct from ours in that we are concerned about the representation and deployment of the beliefs of other agents but not specifically about reasoning about their beliefs. In our context, that task is performed by AI agents that leverage the cognitive models but operate separately (Oguntola, Hughes, & Sycara, 2021).

Several cognitive models have been developed that more closely matched the mechanisms used in our model. Renkema (2018) developed an instance‐based model of the game of “No Thanks” with different levels of proficiency to predict human actions. However, that model is focused on the prediction of aggregate human performance rather than individual differences. Nguyen and Gonzalez (2022) also developed an instance‐based model that learns from observations in a grid world environment and predicts the next step and preferred goal. Predictions are compared to those of a human observer. The model can also predict false beliefs from observation using the Sally–Anne test. Blum, Klaproth, and Russwinkel (2022) developed a cognitive model of anticipation that simulates the mental models of pilots to predict their decisions and behaviors. While our model shares many commonalities with those, we extend the approach by quantifying the workload of those mental representations and using it to optimize interventions.

Cognitive models of the theory of mind have also been developed in other frameworks. Rabkina, McFate, and Forbus (2018) developed a theory of mind model by bootstrapping from language with analogy processes. Bello and Cassimatis (2006) used the Polyscheme cognitive architecture to implement a domain‐general mechanism to represent alternate mental models of the world. Goodman et al. (2006) proposed a rational analysis of children's false belief reasoning using Bayesian causal models of varying complexity.

Other models of theory of mind have also been implemented in the same domain as ours. Rabkina et al. (2020) developed an analogical model of the theory of mind in the stag hunt game and then applied it to goal recognition in Minecraft. Bara, CH‐Wang, and Chai (2021) presented computational models of the theory of mind tasks and proposed applying them to a dataset of collaborative tasks in Minecraft.

## 4 Theory of mind cognitive models in search and rescue task

### 4.1 Search and rescue task in Minecraft environment

**Fig. 2:**
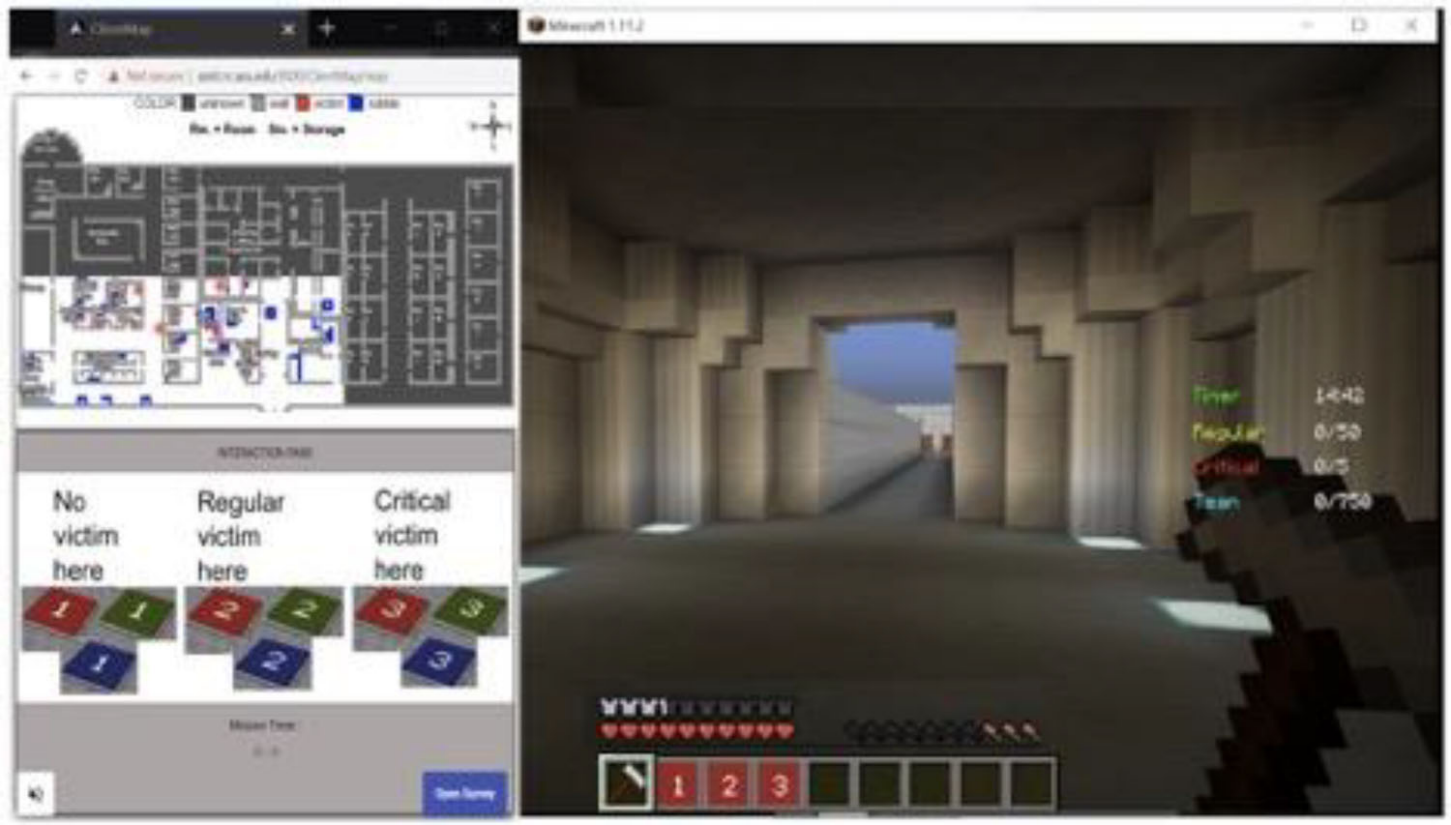
Screenshot of the search and rescue task in the Minecraft environment.

To study the theory of mind in a team environment, a search and rescue task was implemented in the Minecraft environment (Huang et al., 2021). The task involves a team of three players rescuing a sizable number of victims in a large building interior environment (Fig. 2). Rescuing a victim involves a sequence of time‐consuming operations (triaging, evacuating) ultimately resulting in a specified number of points. Victims come in different types, with critical victims resulting in more points but also taking longer to rescue and expiring earlier. Each player has a particular role and characteristics. Players can communicate with each other using natural language over a voice channel as well as through special markers that appear in a shared map of the building environment. Each team of three players makes two 15‐min runs featuring a different building map and distribution of victims. Additional details on the task can be found in the original reference.

### 4.2 Cognitive models as human assistants

**Fig. 3:**
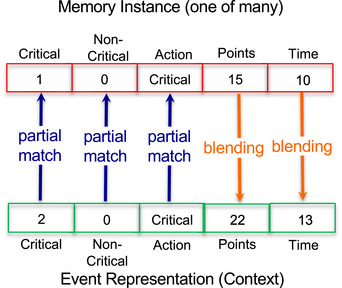
IBL decision process matching context and outputting predicted outcomes.

A basic decision determining task performance involves whether to triage all victims as they are encountered or whether to prioritize critical victims knowing that they will expire halfway through the trial, then triage the non‐critical victims (either previously encountered or still to be encountered) until the end of the trial. Both approaches seem reasonable. Triaging all victims as they are encountered is most efficient in exploring the space but runs the risk of not being able to rescue some critical victims in time. Conversely, prioritizing critical victims will maximize the number triaged by the deadline but might result in time‐consuming backtracking to process previously encountered non‐critical victims and ultimately running out of time before being able to triage them all. Given those tradeoffs and a lack of principled basis for preferring one strategy over another, those decisions are likely to be driven by experience. Therefore, we adopted an IBL modeling approach illustrated in Fig. 3.

The IBL model was implemented in the ACT‐R cognitive architecture, which constitute a theoretical framework that provides primitive computational processes such as memory retrieval, pattern matching, and decision‐making. Experiences in IBL models consist of three parts: (1) a context specifying the value of features relevant to the decision, (2) the action(s) taken, and (3) the resulting outcome(s) and/or their utility. For the triaging decision, the contextual features consist of the number of critical and non‐critical victims in the room. The action is whether to triage all victims as they are encountered or to prioritize critical victims before the deadline. The outcomes are the number of points collected following the triaging decision and the time taken by the process. For each decision, the IBL model uses ACT‐R's blending mechanism (Lebiere, 1999) to generate expected outcomes for each possible decision given previously experienced outcomes. The expected outcome therefore reflects the contextual similarity of the present instance to previous instances weighted by their probability of retrieval and is computed with the following equation:

(1) $$\begin{array}{c} V=\underset{V_{t}}{\mathrm{argmin}}\sum_{i=1}^{n}P_{i}\times Sim{\left(V_{t},v_{it}\right)}^{2} \end{array}$$

The value, V, is the interpolated value based on matching chunks *i*, weighted by their retrieval probability $P_{i}$. The similarity function, $Sim(V_{t},{v_{it})}^{2}$, is used to compare the outcomes in memory chunks $v_{it}$ and candidate consensus values $V_{t}$ and is effectively the loss function to be minimized. In the simplest case, where the outcomes are numerical and the similarity function is linear, the process simplifies to a weighted average by the probability of retrieval.

The retrieval probability $P_{i}$ is derived from a Boltzmann function that is based on the activation strength of instances:

(2) $$\begin{array}{c} P_{i}=\frac{e^{A_{i}/t}}{\sum_{j}e^{A_{j}/t}}\; \end{array}$$

where $A_{i}$ is the activation of instance *i* in memory, and t is the temperature, which was set to a neutral value of 1.0.

The activation *A_i_* of a chunk *i* is determined by the following equation:

(3) $$\begin{array}{c} A_{i}=\ln\sum_{j=1}^{n}t_{j}^{-d}+\mathrm{MP}\;*\sum_{k}\;\mathrm{Sim}\left(v_{k},c_{k}\right)+\varepsilon_{i} \end{array}$$

Recency and frequency are reflected in the power law of practice and forgetting, represented by the first term, where *t_j_* is the time since the *j*th occurrence of chunk *i*, and *d* is the decay rate (set to the default value of 0.5). Similarity between contextual features is reflected in a partial matching process, represented by the second term, where $Sim(v_{k},c_{k})$ is the similarity between the actual memory value and the current value for feature *k* and is scaled by the mismatch penalty (MP), which was set to the default value of 1.0. Similarities for numeric features are linearly scaled from 0.0 (an exact match) to −1.0 (maximum mismatch), whereas symbolic values (the discrete action strategies) are either an exact match (if identical) or maximally different, −10.0. The term $\varepsilon_{i}$ represents transient noise, a random value from a logistic distribution with a mean of zero and variance parameter *s* of 0.1, and introduces stochasticity in retrieval. Model performance was largely insensitive to the specific value of parameters.

The IBL model can perform the task as human players in a purely generative fashion without requiring any training data. The model generates expected outcomes for each action, via blending across previous outcomes. The points/time rate for each action is then computed. A straightforward decision rule is applied: select the action with the highest expected rate. This decision rule is the most common in IBL models and is also compatible with general principles such as the matching law (Poling, Edwards, Weeden, & Foster, 2011). Note that even though the decision rule itself is deterministic, the overall action selection process is stochastic because of the noise in the decision instances stored in memory, leading to fluctuations in expected outcomes. Experienced outcomes then become memory instances factored in future decisions, leading to an amplification of initial experiences in strategy selection (Cranford et al., 2024). To bootstrap the process, one instance for each possible action was created. By default, the expected rate of the strategy of initially focusing on critical victims was twice as high as the rate for the strategy of triaging all victims as they are encountered.

The model saves an additional instance to memory after each action. The new experience associates the features of the situation with the action taken and the resulting outcomes in terms of points gained and time taken. Note that contrary to other IBL models (e.g., Cranford et al., 2021), the expectations generated to make the decision are not themselves stored in memory, a process that can lead to confirmation bias (Lebiere, Jentsch, & Ososky, 2013). Also, the model does not engage in counterfactual reasoning that could result in storing a hypothetical experience for the decision not made. That sampling bias in which only the action taken is subject to have its expectations revised can lead to other cognitive biases such as risk aversion (Lebiere, Gonzalez, & Martin, 2007).

While the model can generate its own behavior, and due to its stochasticity, multiple runs would lead to a distribution in strategy choice (e.g., Cranford et al., 2021), we focus here on its ability to reproduce and predict individual player behavior. To that effect, a process called model tracing, a variant of knowledge tracing used in intelligent‐tutoring systems (Corbett & Anderson, 1995) is used to align the model's instances with the human player's experience. Simply, after the model generates its own decision, it is forced to take the same action as the player and then add an instance reflecting the same outcome in memory. That way, we can compare the accuracy of the model's predictions against the actions of the human player while making sure that they are made from the same basis, that is, the same history of experiences.

We compare the model against two baseline heuristics. The first is the recency heuristic, which predicts that the next user choice will be identical to the previous one. The second is the frequency heuristic, which predicts that the next user choice will be the most frequent one in the past. The ACT‐R base‐level activation equation combines those two factors in the form of power laws. This comparison is therefore an opportunity to determine the relative contribution of the two factors and the value of their combination in the specific form of the cognitive theory.

**Fig. 4:**
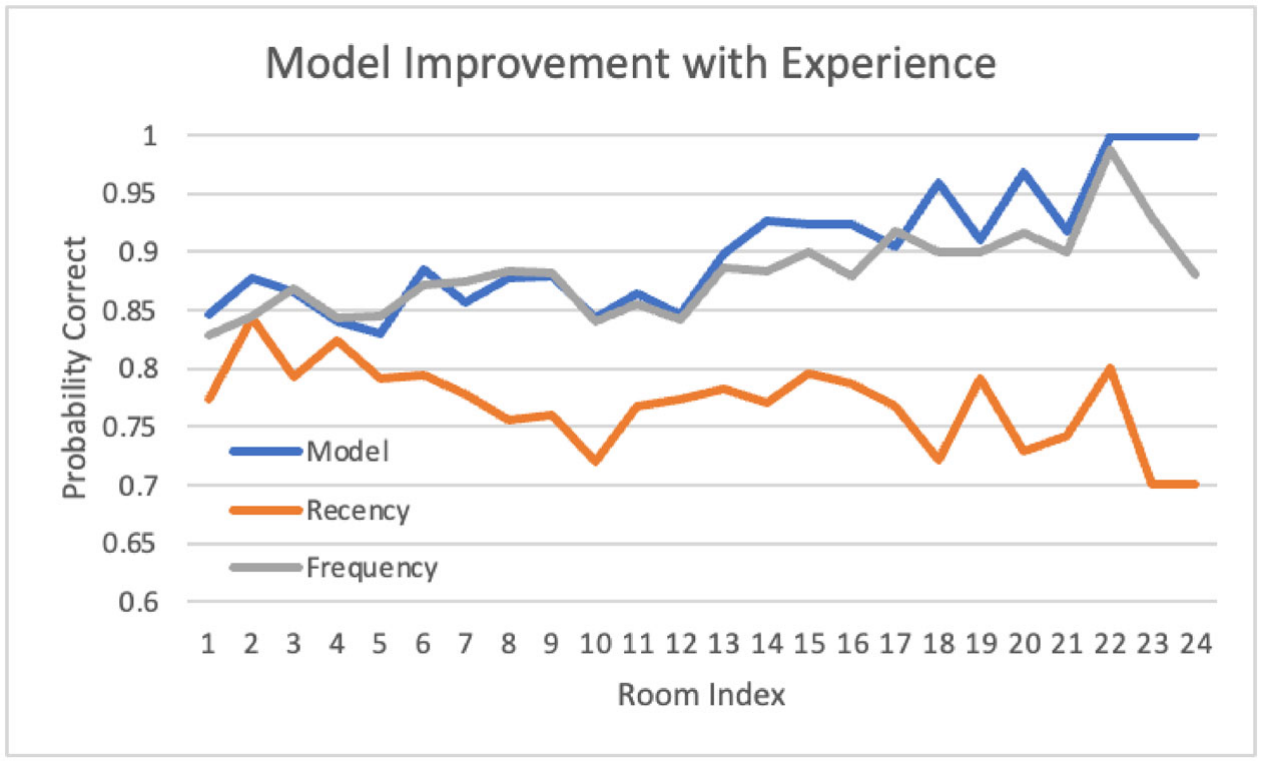
Gradual improvement with experience in the accuracy of predicted decisions.

Fig. 4 presents the accuracy (probability correct) of the model in predicting user decisions as a function of the length of shared experience (number of decisions). Only decisions made before the expiration of critical victims are used since the choice becomes moot after that point. The model improves from about 85% correct initially to 100% accurate after 20 to 25 decisions. It seems to take about 10 to 12 shared experiences for accuracy to significantly improve from its initial level, which could be interpreted as a transition from exploration to exploitation behavior. The frequency heuristic approaches the accuracy of the cognitive model but does not improve nearly as much with experience. The recency heuristic is significantly poorer and does not improve at all with experience since it only reflects the previous trial.

**Fig. 5:**
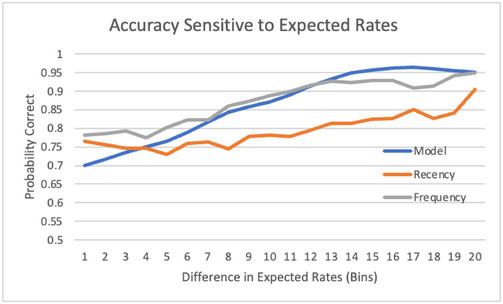
Probability of correct prediction of player move as a function of decision difficulty.

Trial‐to‐trial variations reflect fluctuations in decision difficulty. To quantify the relation between decision difficulty and accuracy, Fig. 5 relates the difference between expected values for the two strategies and the probability of correct prediction of player decision. Prediction accuracy is worse for the closest decisions, then increases gradually until reaching a plateau when differences in expectations between the two strategies are large enough. This relation confirms our hypothesis that players make decisions based on relative expectations for each possible action and that the decision process follows a softmax rule reflecting stochasticity in generated expectations. The relation for the frequency heuristic is not nearly as strong, suggesting that it lacks some sensitivity to the situation. As for experience, the recency heuristic shows very little sensitivity to expectations.

**Fig. 6:**
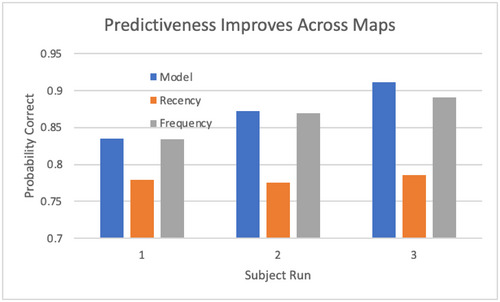
Improvement in prediction accuracy across generalization conditions (maps).

A common issue with models, especially individual models, is whether they generalize across situations. In this study, the main generalization dimension involves runs on three distinct maps involving different configurations of rooms and victims. Fig. 6 demonstrates that model accuracy improves monotonically across maps. The frequency heuristic also improves across maps but not as much as the cognitive model. As expected, the performance of the recency heuristic remains flat.

**Fig. 7:**
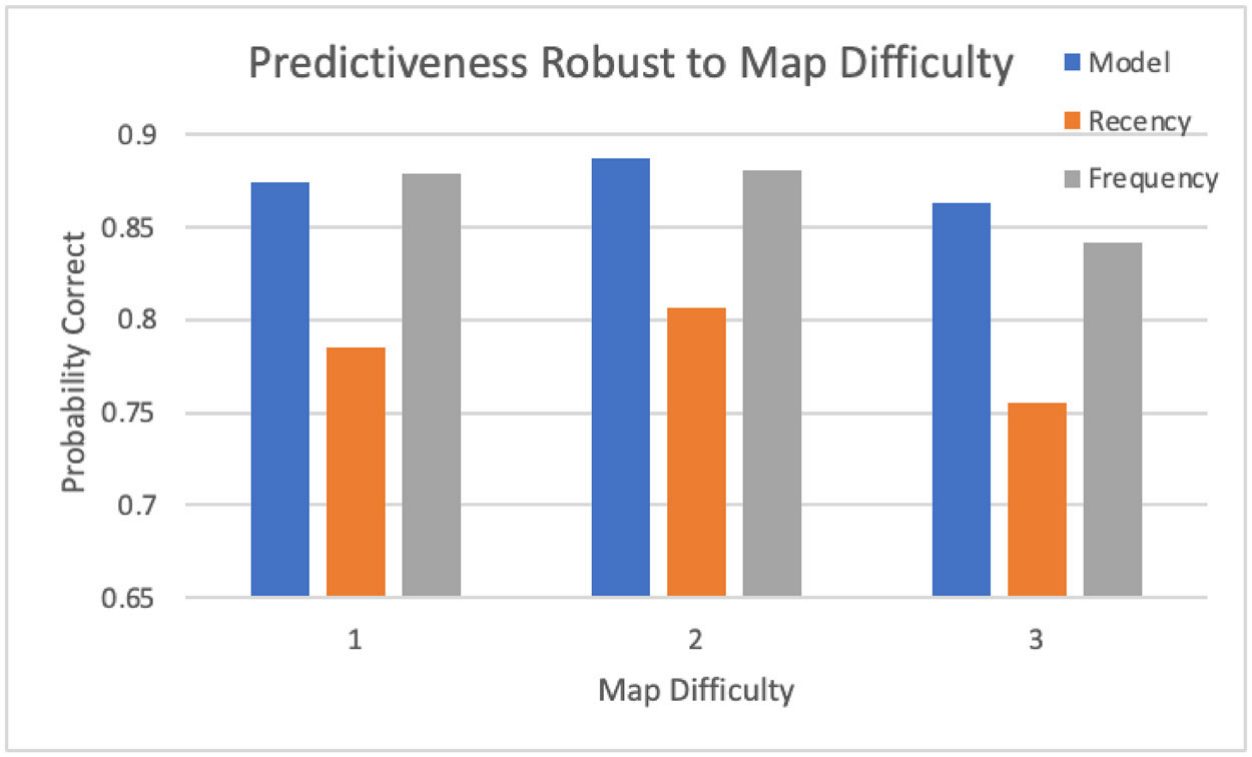
Effect of map difficulty on prediction performance.

Another question is whether prediction accuracy depends on the complexity of the task, specifically the difficulty of the maps, as determined by their complexity. Fig. 7 shows that that is not the case, with no clear effect of map difficulty on prediction accuracy. One explanation is that two factors might work in opposite directions. Easier maps allow for more considered decisions but also more exploration. Conversely, harder maps introduce unpredictable conditions but might lead to simplified strategy choice for the sake of efficiency. Both the frequency and recency heuristics show a stronger effect of map difficulty, lacking the breadth and robustness of the cognitive model.

**Fig. 8:**
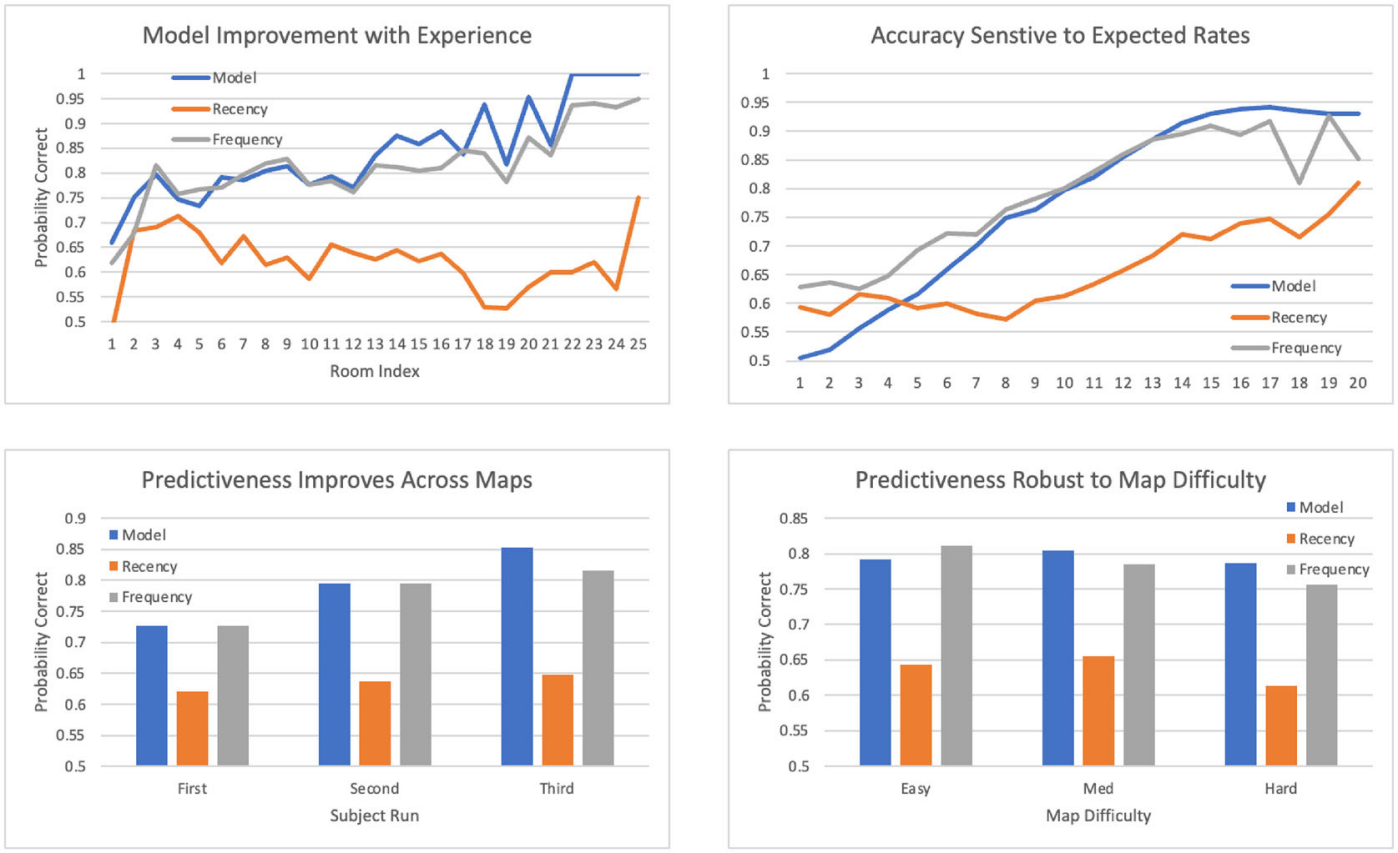
Model performance for unambiguous predictions.

One can wonder why model accuracy starts at almost 85% in Fig. 4 considering that no personalization to the individual player has yet taken place. Two reasons explain the surprisingly high initial performance. One explanation is that some situations (specifically, those featuring only critical victims and no non‐critical victims) cannot differentiate between the two strategies. Therefore, either strategy is counted as an accurate prediction, inflating model accuracy. Fig. 8 presents the same data as Figs. 4, 5, 6, 7 for unambiguous predictions only, confirming that all effects described previously are still present.

**Fig. 9:**
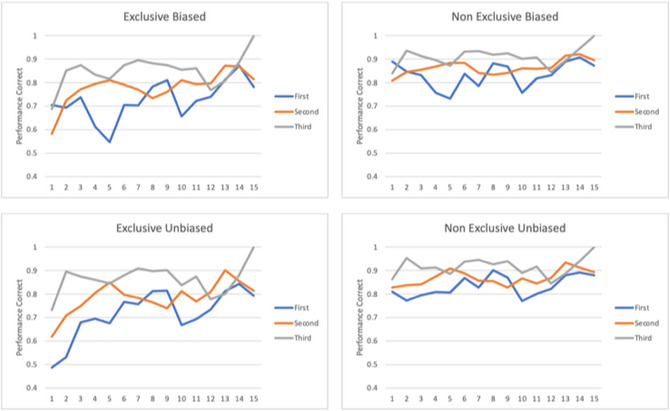
Prediction accuracy curves across levels of experience for all four modeling conditions.

Fig. 8 (top right) shows that performance for two barely distinguishable options is now at chance, but the initial accuracy is lower than 0.85 but still higher than chance (top left). The explanation is that human players have a built‐in preference for the strategy to favor critical victims, presumably because of their higher point value and the emphasis on the triaging deadline, and so does the model since the initial instance for the critical victim strategy has an expected rate twice that of the strategy of triaging all victims. It is not unreasonable to represent in the model the same initial bias exhibited by the human players, but since the origin of that bias is not entirely clear, it is worth asking whether the model could acquire it from experience even if it did not have it initially. To do that, we can initialize the model with the two strategies having equal expected outcomes in their respective instances. Fig. 9 displays the learning curves for all four model combinations for both the original biased initialization and this unbiased initialization, as well as the original full set of (i.e., non‐exclusive) situations and the more exclusive set on unambiguous situations. For each condition, the model prediction accuracy is displayed for each level of experience across all three maps. All previous results are preserved at various quantitative levels in all conditions. The final accuracy for the most difficult (exclusive unbiased) condition shows the same asymptote as the previous conditions despite performing at the chance level at the start of the first run, indicating that the model can learn the individual references of all users in all conditions.

A personalized cognitive model that can learn the specific preferences, patterns, and strategies of individual users can perform any number of roles in human–machine teaming systems. It can serve as a personal recommender that can suggest decisions to lighten the user's cognitive load and improve decision speed. In a more passive role, it can watch over the user and only intervene when it predicts that there is a high probability that the user is about to make a suboptimal decision. Conversely, it can serve as a cognitive twin (Somers, Oltramari & Lebiere, 2020) that replaces the user to perform their role for dangerous or repetitive tasks or in situations where the user is otherwise engaged.

### 4.3 Cognitive models as analytic components

The application of cognitive models as human assistants, recommenders, and stand‐ins requires that the models be able to perform the task to a comparable extent to human users. That places a high bar on requirements since the development of a full‐task cognitive model can be a significant endeavor. An alternative is to derive constraints and predictions from cognitive architectures that would be useful for managing human–machine interaction without requiring the development of a full task model. That approach is compatible with the concept of accountable modeling proposed in conjunction with the ACT‐UP toolkit (Reitter & Lebiere, 2010). Accountable modeling proposes to derive predictions only for aspects of performance that are directly constrained by cognitive theories and mechanisms and treat the other aspects of performance parametrically if at all. Accordingly, the ACT‐UP toolkit provides access to individual mechanisms of the ACT‐R cognitive architecture through a library API in such a way that one can exercise each mechanism independently as it relates to the specific task.

**Fig. 10:**
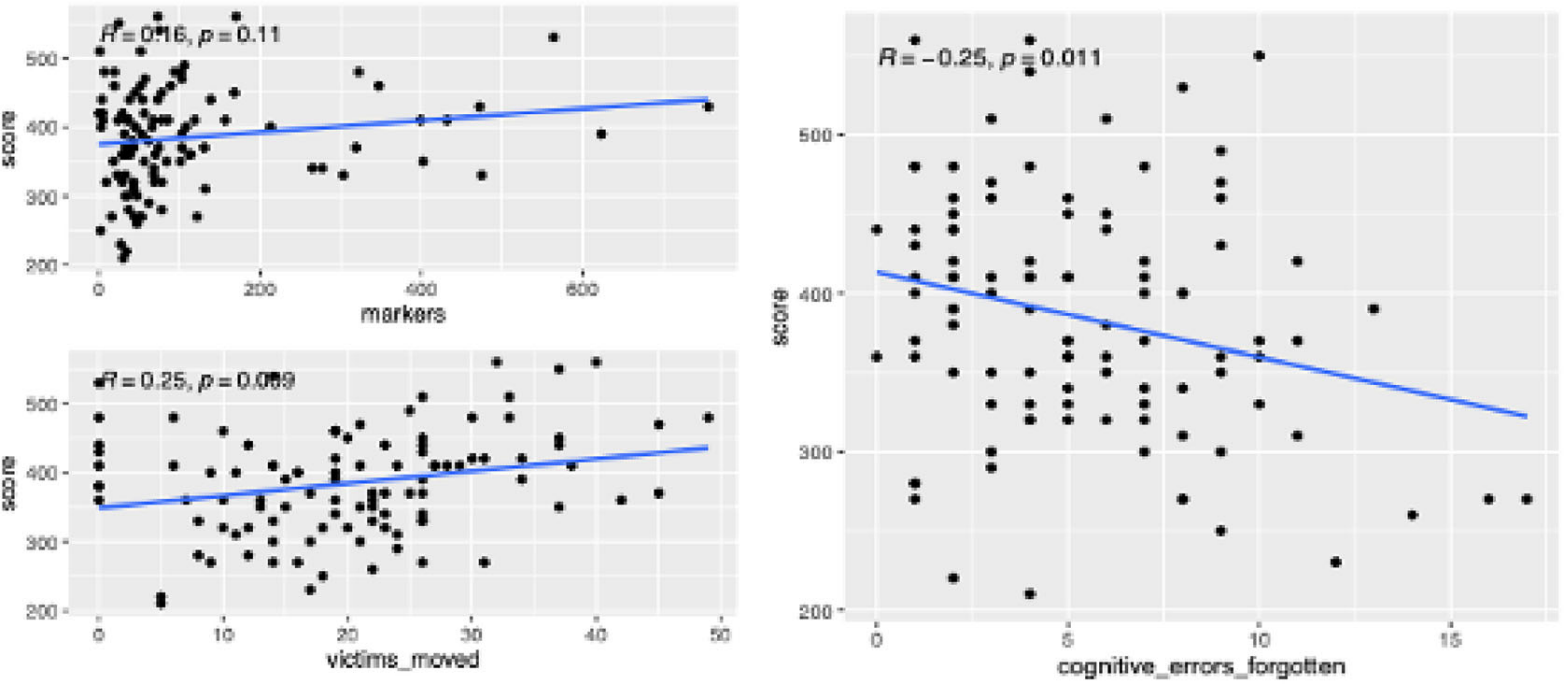
Correlation between score, cognitive load minimizing strategies and cognitive errors.

One key aspect of this task impacted by cognitive limitations is the necessity of remembering some components of the task if one prioritizes some victims over others to achieve a more efficient workflow. Specifically, the location and status of victims that have been discovered but not yet fully processed need to be kept in memory to return to them at some future time during that run. We preregistered the Minimum Cognitive Load Hypothesis that stated that the use of strategies minimizing cognitive load would lead to better team performance (Johnson, Lebiere, & Pirolli, 2021). Fig. 10 (left) displays a positive correlation between two popular strategies for minimizing cognitive load, using markers to indicate the location of victims in a shared map and moving victims to visible locations such as central hallways, and performance score. Conversely, Fig. 10 (right) displays a negative correlation between score and cognitive errors resulting in forgetting to fully process before the end of the trial all the victims that had been discovered.

The effects of keeping future tasks active for efficient processing, that is, prospective memory, have been modeled using activation processes in declarative memory (Lebiere & Lee, 2002). While measures of multi‐channel workload have been mapped in the context of the ACT‐R cognitive architecture to the percentage of utilization of buffers associated with the various architectural modules (Lebiere, 2001), what is needed here is a measure of cognitive load focused on long‐term memory and its activation processes, in particular the process of maintaining memories to keep them from being forgotten. The probability of forgetting memory chunk *i* with activation *A_i_* is given by:

(4) $$\begin{array}{c} P\left(\mathrm{forgetting}i\right)=\frac{1}{1+e^{-\left(\tau -A_{i}\right)/t}} \end{array}$$

where τ is the retrieval threshold, and *t* a scaling parameter reflecting activation noise.

Based on this, Eq. (5) below defines our cognitive load measure, which sums over each relevant memory chunk the odds of forgetting that chunk. Intuitively, each chunk adds a load of 1 when it is just at threshold of being forgotten (i.e., has an activation equal to the retrieval threshold), exponentially smaller when it is stronger in memory and exponentially larger the more its activation falls and it is in danger of being forgotten.

(5) $$\mathrm{Cognitiveload}=\sum_{i=1}^{n}e^{\left(\tau -A_{i}\right)/t}$$

**Fig. 11:**
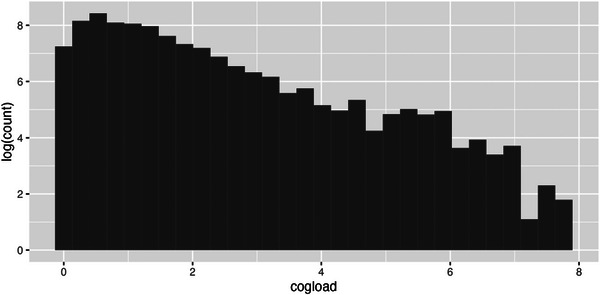
Power law distribution of cognitive load measures (weighted by time) across all runs.

To apply our cognitive load formula to a search and rescue task scenario, we need to define which events need to be remembered and forgotten. A victim, with its location and current status, is added to memory when it is first encountered. It receives an additional rehearsal, leading to an increase in activation, at each step of processing such as triaging or moving the victim or placing a marker block at its location, and is finally forgotten once it is evacuated. Fig. 11 displays the distribution of cognitive load measures over all runs by human teams. If one interprets cognitive load as a scale‐free measure (as is memory activation, defined as log odds of retrieval), then we see that it closely follows a clear power law distribution typical of many human environments (Newell & Rosenbloom, 1981; Anderson & Schooler, 1991), an indication that our measure of cognitive load reflects the cognitive environment.

**Fig. 12:**
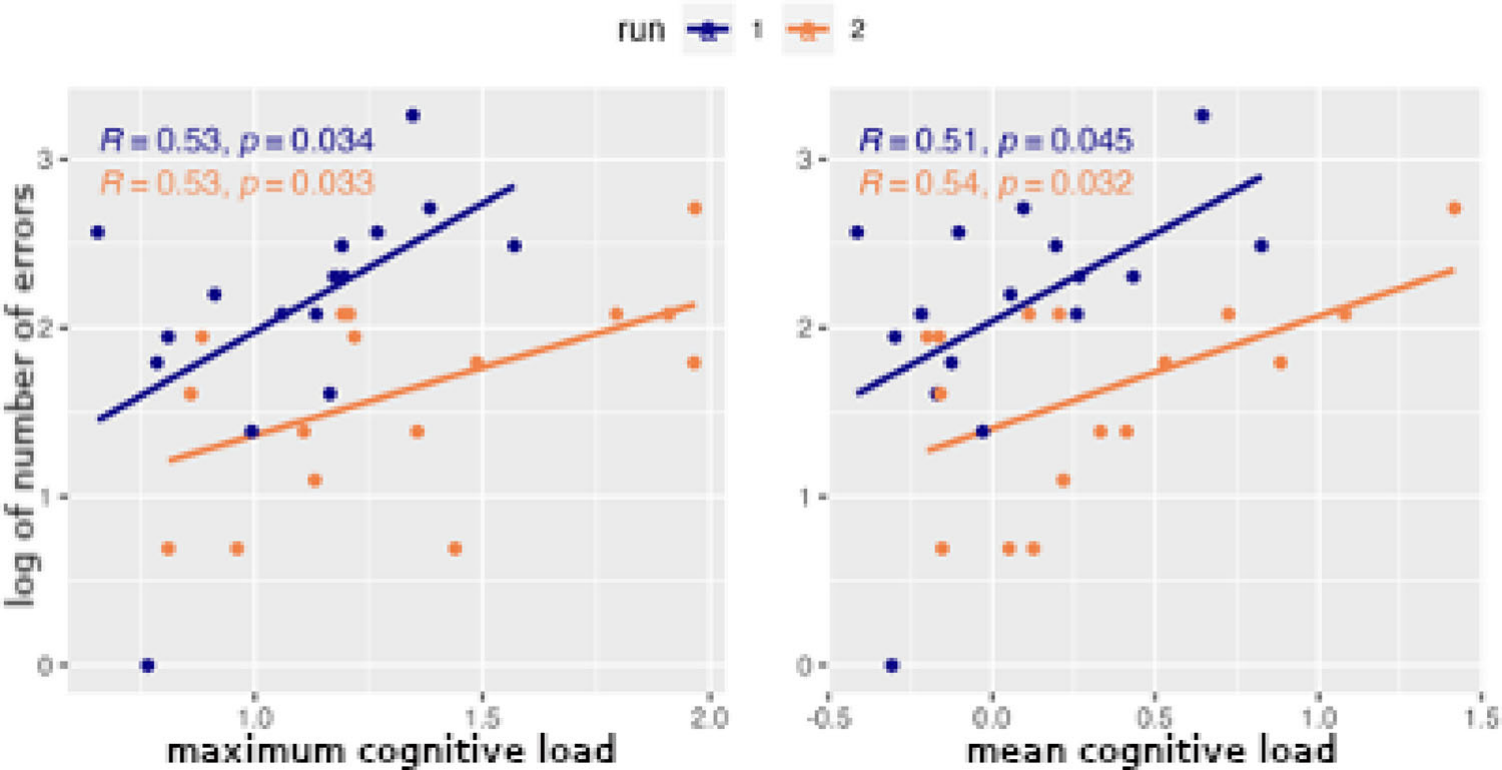
Correlation between maximum (left) and mean (right) cognitive load and number of errors.

Now that the cognitive load measure has been formally defined, we hypothesized that it would be strongly correlated with the number of related errors such as forgetting to fully process victims that had been discovered. Fig. 12 displays the correlation between cognitive load and the number of errors during a run. The correlation is quite strong for both maximum cognitive load (reflecting the highest pressure during a run) and mean cognitive load (reflecting sustained mental load). Also, the correlation remains as high for the second run of each team as the first, indicating that the relation between cognitive load and errors is retained even as the overall number of errors decreases with practice.

**Fig. 13:**
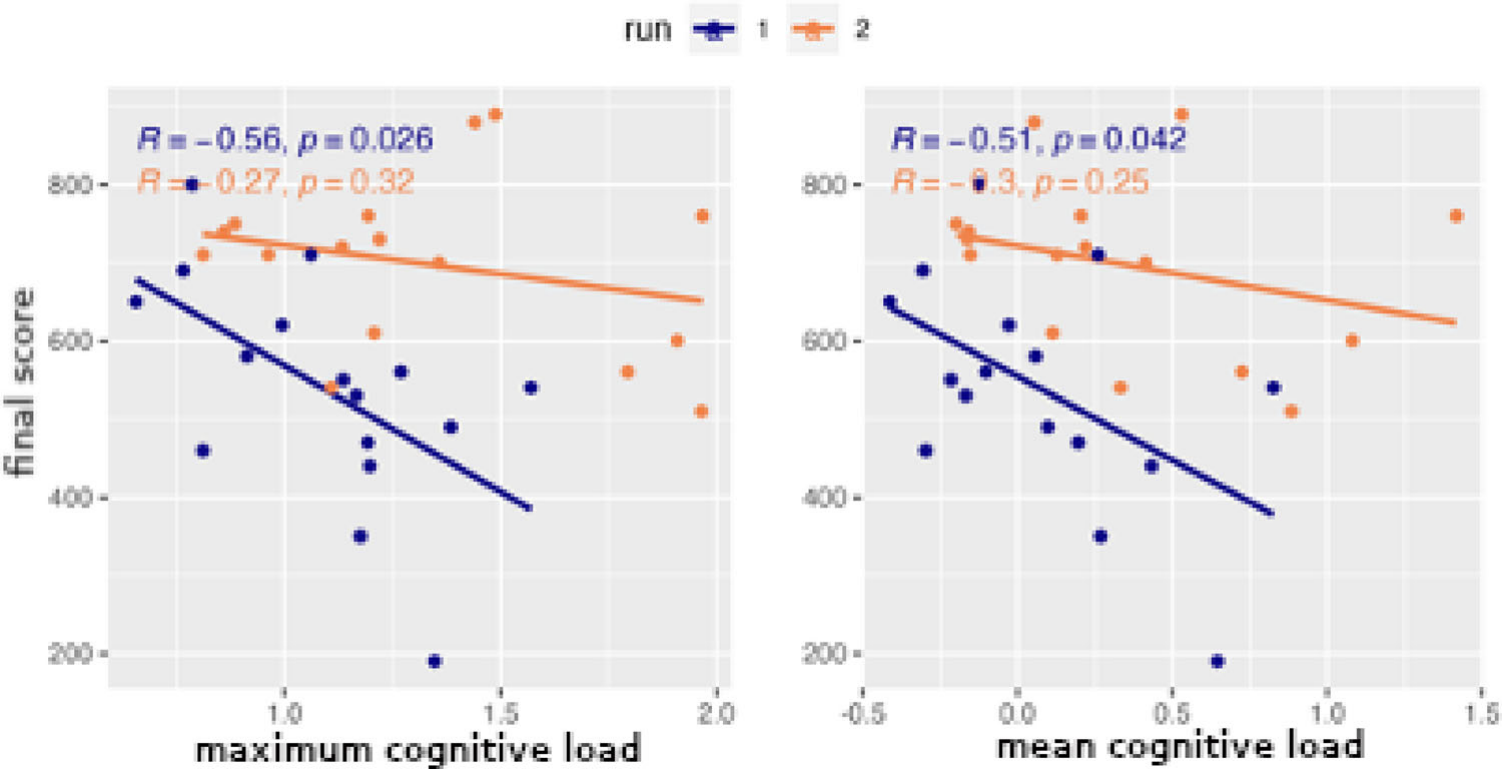
Correlation between maximum (left) and mean (right) cognitive load and final score.

Fig. 13 displays the correlation between measures of cognitive load during a run and performance in terms of final score. The correlation for both mean and maximum cognitive load is comparable to the previous result for the first run but is cut roughly in half for the second run. One can attribute the decrease to the increasing prominence of other factors in determining performance, such as improved individual skills and teaming strategies.

**Fig. 14:**
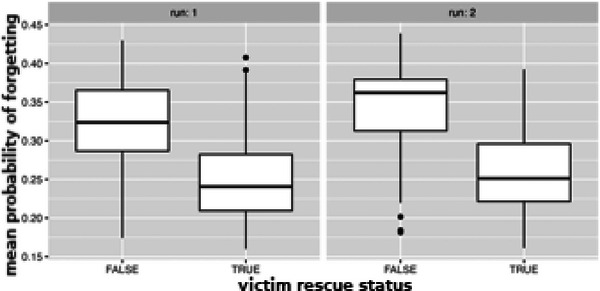
Mean probability of forgetting depending upon victim rescue status across runs.

Finally, one can evaluate how accurately the cognitive load model can predict not just overall errors and scores but the probability of forgetting a specific victim. Fig. 14 computes the mean probability of forgetting depending on whether the victim was ultimately processed or not. Victims who were not fully processed had a significantly higher mean probability of forgetting across the run, and that relation holds across runs (practice).

**Fig. 15:**
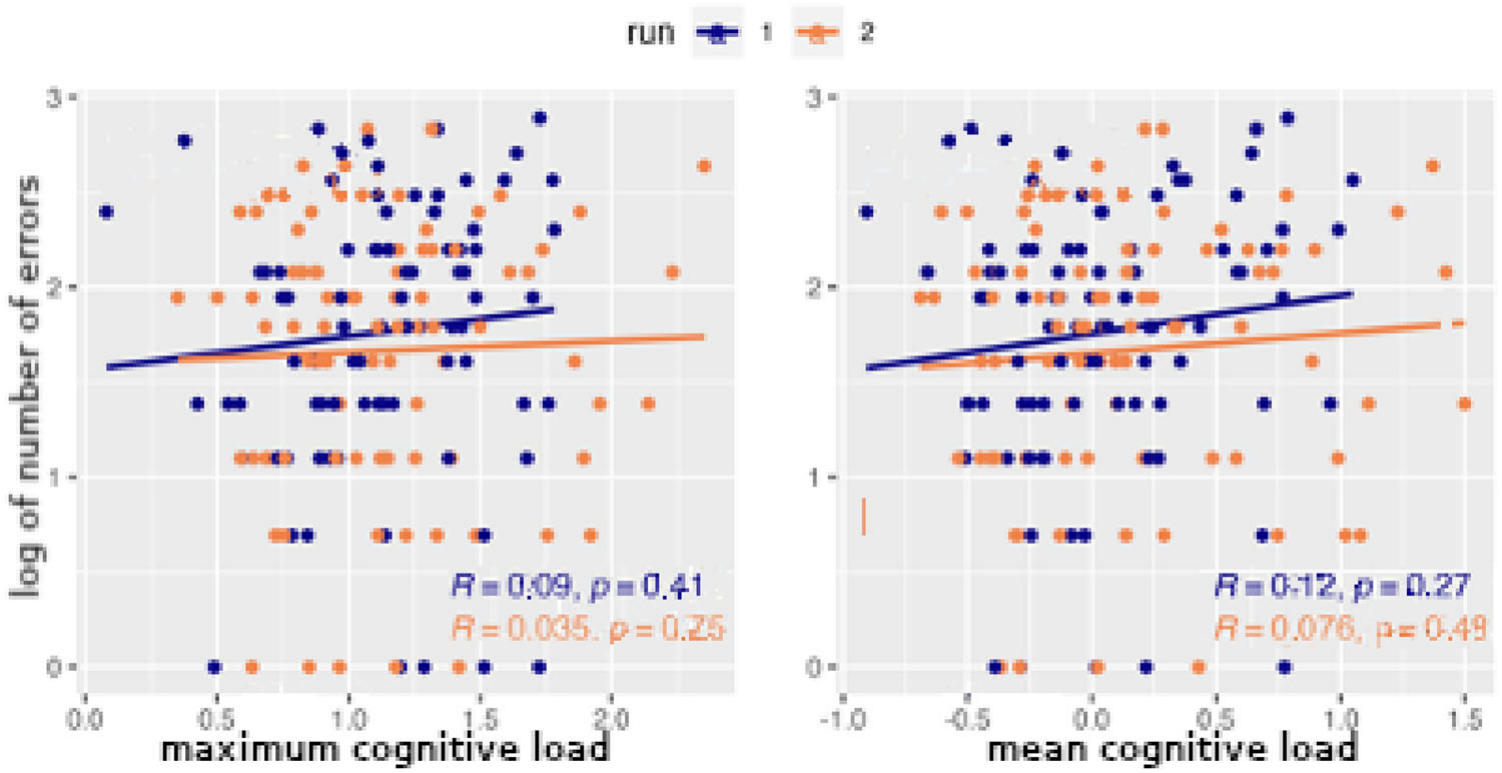
Correlation between maximum (left) and mean (right) cognitive load and number of errors for runs featuring an artificial social intelligence agent.

Once predictive measures such as cognitive load and probability of forgetting can be computed in real time, they can serve as *analytic components* in artificial social intelligence (ASI) agents that can use them as indicators to drive when and how to intervene to improve teamwork in human players. Those interventions can take the form of reminders about tasks to be performed (such as a victim still to be processed) or suggestions for collaboration (how to schedule joint tasks). Our cognitive load analytic component was integrated in a number of ASI agents, some of which are described in this special issue. Fig. 15 describes the correlation between cognitive load and number of errors, similar to Fig. 12. While some errors remain, the correlation has largely disappeared, suggesting that the cognitive load analytic component was effectively used by the ASI agents to prevent errors driven by cognitive load.

## 5 Discussion

**Fig. 16:**
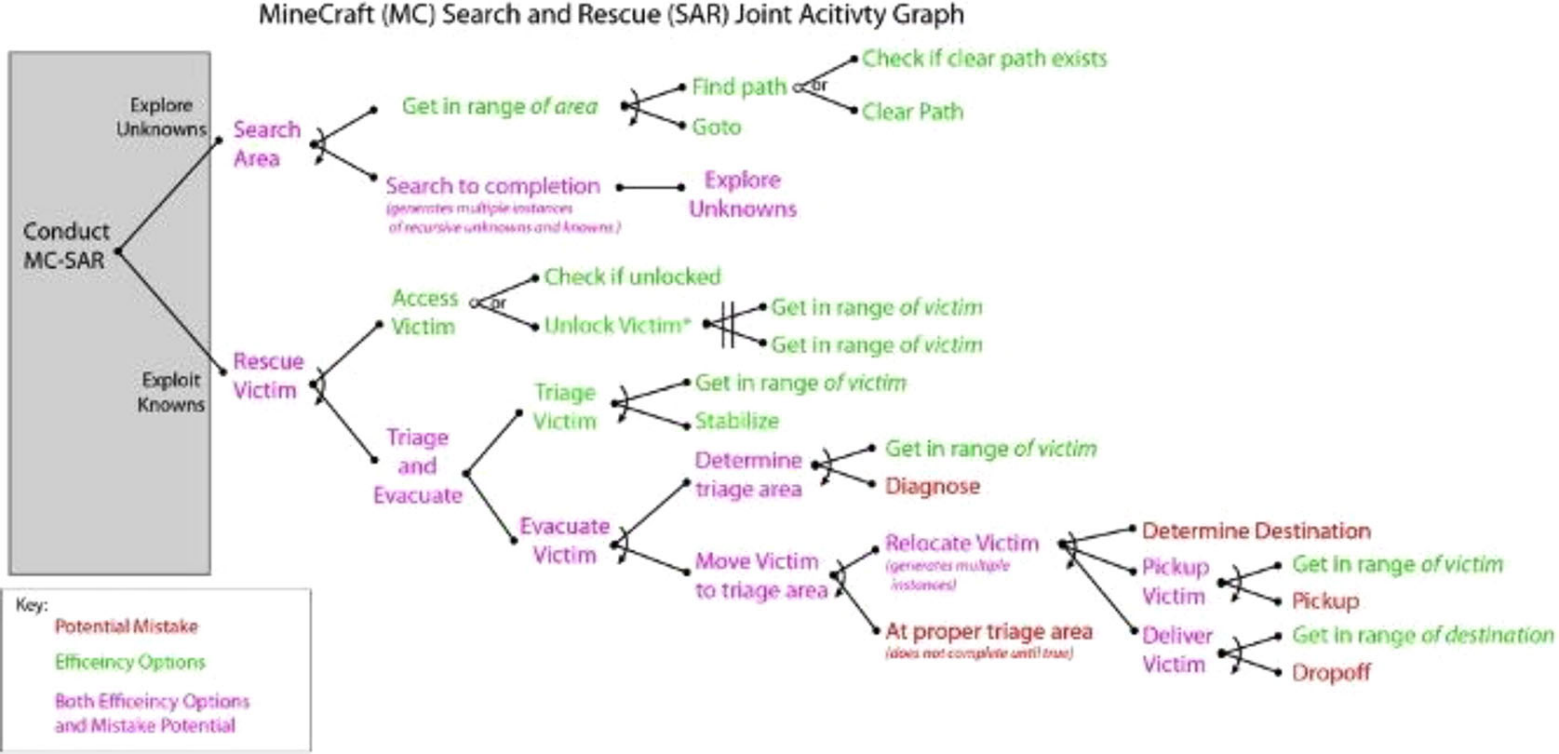
Joint activity graph.

The cognitive models that we presented focused on individual human performance such as decisions regarding triaging strategies or cognitive load involved in remembering contextual information. However, they did not focus on teamwork per se. Previous work (Best & Lebiere, 2006; Lebiere et al., 2013) regarding the modeling of teams involved representations and execution of joint plans of action. As a more general and scalable approach at representing team activities, we have developed a representation called joint activity graph (JAG) that capture both task and cognitive dependencies between individual actions (Fig. 16). In future work, we aim to use those graphs as knowledge structures in cognitive models that could integrate teamwork in both approaches that we described. JAGs could be used in cognitive models that perform tasks in concert with human and machine teammates. They could also support analytic components that reflect cognitive factors involved in teamwork as well as taskwork.

## 6 Conclusion

We described two complementary ways in which cognitive architectures can provide a MToM to facilitate collective human–machine intelligence. The first approach is to develop cognitive models of the tasks performed by human users, then personalize them to the preferences, knowledge, and skills of individual users. These individualized models can then serve a number of roles, including a personal assistant that takes over when needed, a recommender that suggests most fruitful actions, and a supervisor that anticipates and prevents mistakes. The second approach is to use cognitive mechanisms to provide analytic components that do not perform entire tasks but provide quantitative insights into the cognitive processes of human teammates. ASI agents can then use those indicators to intervene with the goal to facilitate collaboration between human teammates. Future work will involve incorporating teamwork in both approaches to develop cognitive models that can advise and replace humans in mixed human–machine teams as well as analytic components that can optimize artificial agents to the cognitive workings of their human teammates.

## Note

1 The ACT‐R simulation environment, including source code, manual, and tutorial units, and hundreds of papers and workshop proceedings are available on the web at: http://act‐r.psy.cmu.edu/.
